# Manganese and IL-12 treatment alters the ovarian tumor microenvironment

**DOI:** 10.18632/aging.205361

**Published:** 2024-01-03

**Authors:** Yan Xu, Xin Huang, Xiao-Cui Nie, Yan-Song Liu, Yang Zhou, Ju-Min Niu

**Affiliations:** 1 Department of Gynecology, Shenyang Women and Children’s Hospital, Shenyang 110000, China; 2 Department of General Practice Medicine, Shengjing Hospital Affiliated to China Medical University, Shenyang 110000, China; 3 Department of Obstetrics and Gynecology, Shengjing Hospital Affiliated to China Medical University, Shenyang 110000, China

**Keywords:** metal immunotherapy, Mn^2+^, IL-12, macrophage, ovarian tumor

## Abstract

Metal immunotherapy is a novel adjuvant immunotherapy. Mn^2+^ can activate STING—a type I IFN response protein—that promotes innate immunity and increases anti-tumor activity by promoting macrophage phagocytosis. IL-12, a cytokine that increases the antigen-presenting ability to promote effector T-cell activation, has potent antitumor activity, albeit with severe adverse effects. In this study, we observed that the combination of Mn^2+^ and IL-12 has a better antitumor effect and possibly reflects a better safety profile, providing a novel approach and theoretical basis for safe and rapid cancer treatment.

## INTRODUCTION

Immunotherapy is revolutionizing cancer treatment [1–3]. However, limited response rates have been attributed to the poor antitumor immunity of patients with “cold” tumors, which are characterized by the formation of an immunosuppressive microenvironment facilitated by immune cells in the tumor microenvironment (TME), which promotes tumor growth [4].

In solid tumors, macrophages can account for up to 50% of all immune cells and are one of the most abundant cells in the TME [5]. Macrophages are classified into M1 (classically activated) and M2 (alternatively activated). M1 macrophages exert proinflammatory effects that slow tumor progression. Tumor-associated macrophages (TAMs), mainly M2 macrophages, promote tumor invasion by producing matrix metalloproteinases and promote tumor angiogenesis by releasing platelet-derived growth factor and vascular endothelial growth factor [6], thereby contributing to the formation of an immunosuppressive TME. Infiltrating CD8^+^ T-cells in the TME can recognize and destroy tumor cells. Upon professional antigen-presenting cell stimulation, CD8^+^ T cells proliferate and differentiate into effector cells, namely, cytotoxic T lymphocytes [7]. Macrophages internalize and present tumor-associated antigens to trigger the expansion of tumor-specific CD8^+^ T cells [8]. Activated CD8^+^ T cells destroy tumor cells by releasing perforin and granzymes or by producing Fas ligand (FasL) and tumor necrosis factor-related apoptosis-inducing ligand (TRAIL). Therefore, promoting macrophage polarization towards the M1 type or enhancing CD8^+^ effector T cells through antigen presentation is considered a rational tumor treatment strategy [9].

The stimulator of the interferon genes (STING) pathway plays a vital role in the initiation of antitumor immunity and the transformation of “cold” to “hot” tumors [10, 11]. Emerging evidence has demonstrated the crucial roles of metal ions in immune regulation [12], including T cell activation (Ca^2+^) [13], pathogen–host interactions (Mg^2+^ and Mn^2+^) [14, 15], and cGAS-STING signaling (Mn^2+^) [15].

Manganese is a trace element involved in various physiological activities, including reproduction, development, energy metabolism, and antioxidant defense [16]. Mg^2+^ also regulates innate immunity by sensitizing the STING pathway [17]. As a natural and readily available STING agonist, Mn^2+^ may be a promising immunotherapeutic adjunct. Mn^2+^-deficient mice exhibited higher tumor burden and fewer tumor-infiltrating CD4^+^/CD8^+^ T cells than those in normal mice [18]. In addition, Mn^2+^ promoted the antigen-presenting ability of macrophages, thereby enhancing the activation of CD8^+^ T cells and natural killer (NK) cells [19], which may be associated with Mn^2+^ promoting the phagocytosis of cancer cells by macrophages [20].

IL-12, a proinflammatory cytokine with potent tumor suppression activity, is a promising candidate for combination immunotherapy. IL-12 directly supports the sustained cytotoxic activity of T-cells, improves antigen presentation, mitigates antigen-negative escape, and remodels endogenous immunosuppressive cells within the TME [21]. In preclinical models, IL-12 has demonstrated promising results in promoting tumor clearance by enhancing T and NK cell expression [22]. However, systemic administration of IL-12 increases the risk of complications associated with IFN-γ toxicity, which limits its use as an immunotherapy [23].

Compared with the effects of monotherapies, we hypothesized that an Mn^2+^- and IL-12-containing cocktail therapy would have better therapeutic effects on solid tumors by simultaneously enhancing innate and adaptive immunity and overcoming multiple immunosuppressive factors. In the present study, we explored the critical roles of Mn^2+^ and IL-12 in macrophage activation *in vitro*. Furthermore, we investigated the combined antitumor effects of Mn^2+^ and IL-12 in a mouse tumor model.

## MATERIALS AND METHODS

### Cell lines

The ID8 cell line (a mouse ovarian epithelial cell line) was purchased from the American Type Culture Collection (ATCC, Manassas, VA, USA). ID8 cells were cultured in Dulbecco’s modified Eagle medium (DMEM) supplemented with 10% FBS and 1% insulin transferrin selenium (ITS).

### Isolation of bone marrow-derived macrophages (BMDMs)

Primary mouse BMDMs were isolated, as previously described, with some modifications [24]. Femurs were removed postmortem, and the bone marrow was flushed and collected by centrifugation at 400 × *g* for 5 min at 4°C. The cell pellet was resuspended in 1 mL RBC lysis buffer and centrifuged at 400 × *g* for 5 min. The remaining cells were resuspended in DMEM containing 20 ng/mL GM-CSF (HY-P7361, MedChemExpress, Shanghai, China) and 10% fetal bovine serum (FBS). On day 3, half of the medium containing 20 ng/mL GM-CSF was replaced, and on day 6, the cells were harvested for experiments.

Mouse T cells were isolated from murine spleens using a mouse T cell isolation kit (100-0204, Stemcell Technologies, Vancouver, Canada) and subsequently stimulated with anti-mouse CD3/CD28 magnetic beads for 24 h. Activated T cells were cultured in RPMI 1640 medium containing 10% heat-inactivated FBS, 2-mercaptoethanol (50 μM), recombinant human IL-2 (100 U/mL; Shanghai Huaxin High Biotech, Shanghai, China), penicillin (100 U/mL), and streptomycin (0.1 mg/mL).

### Western blot analysis

Cells were washed thrice with PBS and lysed with lysis buffer (P0013B, Beyotime Biotechnology, Shanghai, China) containing a protease inhibitor cocktail (HY-19303A, MedChemExpress) and phosphatase inhibitor cocktail (HY-K0023, MedChemExpress). Protein concentrations were determined using a BCA Protein Assay Kit (P0010S; Beyotime Biotechnology). Proteins were diluted with loading buffer (P0015L, Beyotime Biotechnology) and heated to 95°C for 10 min. Denatured proteins were stored at −20°C. For Western blotting, equal amounts of protein (calculated according to concentration) were electrophoresed on 10% sodium dodecyl sulfate-polyacrylamide gel, transferred to nitrocellulose membranes, blocked with 5% skim milk for 2 h at room temperature, and incubated with the corresponding primary antibodies against STING (1:1,000, #13647; Cell Signaling Technology, Boston, MA, USA), IRF3 (1:1,000, #4302; Cell Signaling Technology), P-IRF3 (1:1,000, #29047; Cell Signaling Technology), P-TBK1 (1:100, #5483; Cell Signaling Technology), and TBK1 (1:500, #38066; Cell Signaling Technology) overnight at 4°C. Membranes were washed three times for 15 min each with Tris-buffered saline containing TBST and incubated with HRP-linked peroxidase-linked anti-rabbit immunoglobulin G (IgG, 1:1,000, s0001; Affinity Biosciences, Jiangsu, China) for 1 h at room temperature. After three washes with TBST, the protein bands were wetted with Immobilon Western chemiluminescent HRP substrate (Merck Millipore, Darmstadt, Germany) and detected using a Las 4000 luminescent image analyzer (Fujifilm, Tokyo, Japan).

### Quantitative RT-PCR analysis

Total RNA was isolated using TRIzol reagent (RR820A; Takara Bio, Beijing, China) according to the manufacturer’s instructions. We converted 1 μg of total RNA to cDNA using random primers and Superscript III reverse transcriptase (RR047A; Takara Bio). PCR, using gene-specific primer sets, was performed with LightCycler^®^ Quantitative real-time PCR SYBR Green master mix on 96-well reaction plates with an ABI 7500 system, and mRNA expression was calculated as the cumulative index (2^–ΔΔCt^). The primer sequences used in the real-time PCR analysis are presented in Table 1.

**Table 1:** The primer sequences used in the real-time PCR analysis.

**Oligo name**	**Forward primer (5′–3′)**	**Reverse primer (5′–3′)**
**CD80**	CCC CAG AAG ACC CTC CTG ATA G	CCG AAG GTA AGG CTG TTG TTT G
**CD86**	TCA GTC AGG ATG GGA GTG GTA	AGG TAG GAA TGG CTC TTG GAT
**IRF-7**	GGC TGG AAA ACC AAC TTC C	GCC TCT GCC TCA GTC TGG T
**IFN-γ**	GAA CTG GCA AAA GGA TGG TGA	TGT GGG TTG TTG ACC TCA AAC
**Perforin**	CAG ACA GAT GGA AAA GGG AGA T	AGA ATG GCG GAG GGC TTA G
**IL12RB1**	GGA CCA GCA AAC ACA TCA CC	TTC AAC GCA GCA GC CAT CAC
**GAPDH**	GGA GCC AAA AGG GTC ATC ACT C	GAG GGG CCA TCC ACA GTC TTC T

### Evaluation of metal ions in modulating type I IFN responses *in vitro*

To screen metal ions that regulate the STING/IFN-I response, we seeded 100,000 BMDM cells per well in 96-well plates with 200 μM of metal ions, including Mg^2+^, Ca^2+^, and Mn^2+^. After incubation for 24 h at 37°C and 5% CO_2_, the supernatant was collected for IFN-β ELISA assay (E-EL-M0033c, Elabscience, Wuhan, China).

### Phagocytosis assay

For synchronized phagocytosis, adherent macrophages (BMDMs) and pHrodo Red-labeled tumor cells (ID8) were isolated using 0.25% trypsin EDTA and resuspended in antibiotic and serum-free DMEM at a density of 1.5 × 10^6^ cells/mL. The assay was performed in 96-well plates with 200 μL of the cell solution per well. The tumor cell-to-macrophage volume ratio was 3:1, and the plates were placed on ice for 15 min. The plates were centrifuged at 150 × *g* for 5 min at 4°C to facilitate contact between the tumor cells and macrophages. Phagocytosis was induced by replacing the supernatant with medium preheated to 37°C with or without Mn^2+^ and then incubating the plates at 37°C and 5% CO_2_. After the indicated time periods, the cells were placed on ice to stop phagocytosis, washed, and resuspended in PBS for flow cytometry. Typical forward- and side-scatter gates were used to exclude the dead cells and aggregates. A total of 3 × 10^4^ engulfment events were identified by pHrodo Red and GFP double-positive cells. The phagocytic index was calculated as the total number of phagocytes that were pHrodo Red and GFP double-positive/total number of macrophages (number of GFP-positive cells).

### T-cell stimulation experiments

ID8 tumor lysates were prepared by freeze-thaw cycles as previously described [25]. Tumor lysates of ID8 were prepared and co-cultured with mouse BMDMs treated with different agents. The cell lysates were then washed with PBS, and the cleaned BMDMs were re-added to the medium and co-cultured with T cells.

### Mouse tumor models

We explored the antitumor effects of Mn^2+^ in combination with IL-12 in a mouse tumor model. All mice were subcutaneously injected with 1 × 10^7^ live ID8 cells and treated once every other day for 12 days with 5 mg/kg Mn^2+^ (intranasal or intratumoral administration) and 50 U IL-12 (intraperitoneal injection). Tumor growth was monitored, and tumor volume was calculated as follows: volume = 0.5 × length × width^2^. The mice were sacrificed when the tumor volume exceeded 2500 mm^3^ or at the end of the experiment. We sacrificed the mice using cervical dislocation method after anesthesia (pentobarbital 50 mg/kg).

### Immunohistochemical (IHC) and immunofluorescence (IF) staining

Tissue sections (5-μm thick) were placed on a slide and dried in the incubator at 37°C. After xylene dewaxing, the slices were immersed in 50%, 70%, and 80% ethanol for 2 min each and in eosin for 5 s. The specimens were then routinely dehydrated, made transparent, and fixed. The sections were washed with water, the antigen was heat repaired, the primary antibody was incubated, and the secondary IgG was incubated with appropriate biotin (1:1,000, #S0001, Affinity Biosciences, Wuhan, China). The slices were stained with hematoxylin for 5 min, rinsed with tap water for 3 min, rinsed with 1% hydrochloric acid for 2 s, rinsed with tap water for 2 min, and then sealed by gradient dehydration. For IF staining, the specimens were covered with a fluorescent-labeled antibody and stored in an enamel box for 30 min. Examination of co-stained sections and images were collected using a light microscope (Olympus, Tokyo, Japan). The corresponding primary antibodies were against CD80 (ab254579, Abcam, Cambridge, UK), CD86 (ab119857, Abcam), F4/80 (ab60343, Abcam), iNOS (ab178945, Abcam), CD4 (ab183685, Abcam), CD8 (ab237723, Abcam), CD3 (ab135372, Abcam), CD44 (ab243894, Abcam), and Il-12R (ab282729, Abcam).

### TUNEL assay

DNA fragments were analyzed by the transferase dUTP nick-end labeling (TUNEL) method. After dewaxing, the samples were rehydrated using graded ethanol and immersed in PBS containing 4% formaldehyde for 20 min. The samples were subjected to click and *in situ* apoptosis detection, TUNEL analysis, and Alexa Fluor 488 dye staining (c10617; Thermo Fisher Scientific, Waltham, MA, USA). Nuclei were stained with VECTASHIELD installation medium (h-1400, Vector Laboratories, Newark, CA, USA). Ki-67 was used as a marker of proliferation with red fluorescence. TUNEL-positive cells exhibited green fluorescence. Immunofluorescence images were obtained using a fluorescence microscope.

### Bioinformatics analysis

The Gene Expression Omnibus (GEO) database (http://www.ncbi.nlm.nih.gov/geo) was searched for datasets related to the gene dataset of Mn^2+^ on myeloid cells (GSE77216). The expression matrix was analysed using intergroup analysis and principal component analysis (PCA). Differential analysis was performed using the R package “limma” (3.5.3) from the Bioconductor project. GO (Gene Ontology) and KEGG (Kyoto Encyclopedia of Genes and Genomes) pathway enrichment analysis of differential genes using the Clusterprofiler package, the biological significance was explored by the results visualization, and the significance threshold was set at *P* < 0.05. Visualisation of the results was done using R.

### Data analysis

All statistical analyses were performed using GraphPad Prism 8.0 (GraphPad Software, La Jolla, CA, USA). For quantitative data, continuous variables were expressed as the mean ± standard deviation (x ± s). One-way ANOVA was used to compare groups with Tukey’s post hoc test for pairwise comparisons. ANOVA and Mann–Whitney *U* tests were used to compare data among different groups. For all tests, significance was set at a two-tailed *P*-value of < 0.05.

### Availability of data and materials

All the data and materials that are required to reproduce these findings can be shared by contacting the corresponding author on reasonable request.

## RESULTS

### Mn^2+^ enhances macrophage STING activity and antitumor capacity *in vitro*

**Figure 1:**
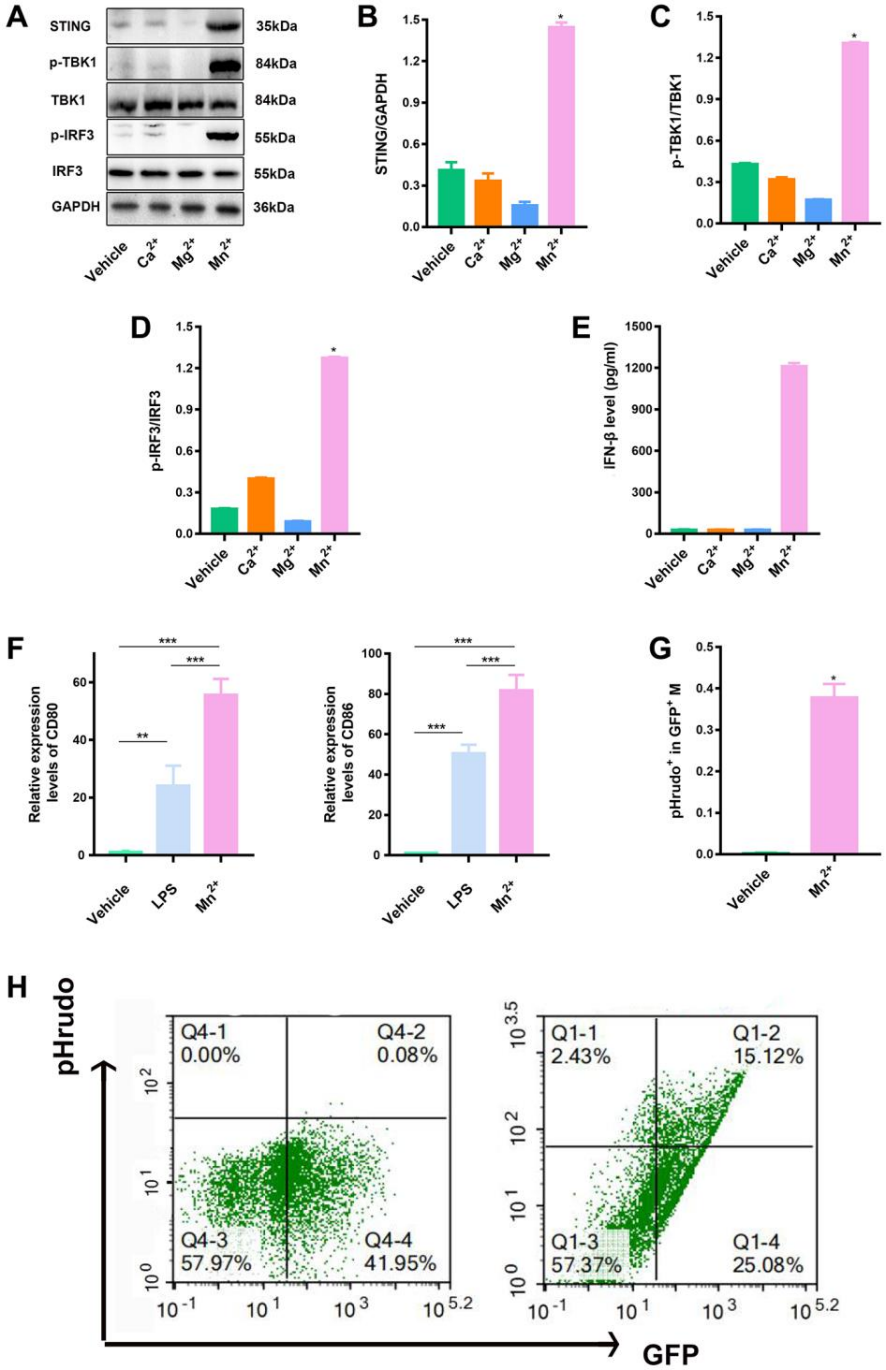
**Mn^2+^ enhanced macrophage STING activity and antitumor capacity *in vitro*.** (**A**–**D**) Macrophage STING activity related to treatments with different metal ions; the marker proteins in downstream pathways were immunoblotted. (**E**) Macrophages were incubated with different metal ions for 24 h, and the response to IFN-β secretion was quantified. (**F**) qRT-PCR analysis of CD80 and CD86 gene expression. (**G**, **H**) To quantify the phagocytic sis of macrophages to tumor cells by flow cytometry, we co-cultured BMDM with pHrodo-labeled tumor cells (ID8) for 24 h, and further observed the effect of macrophages on tumor cells by flow cytometry. One representative experiment is presented from at least three independent experiments, each performed in triplicate. Error bars represent SEM. Data were analyzed by unpaired *t*-test. Abbreviation: NS: not significant. ^*^P < 0.05, ^**^P < 0.01, ^***^P < 0.001.

We examined the activation of the STING type I IFN pathway by treatment with various metal ions (e.g., Ca^2+^, Mg^2+^, and Mn^2+^) to identify novel metal immunotherapy that enhances STING activity. Maximal transcription of IFN-I genes depends on the formation of an enhanceosome containing phosphorylated IRF3 and TBK1. Mn^2+^ effectively elevated the levels of STING along with phosphorylated TBK1 and IRF3 (Figure 1A–1D) and type I IFN (IFN-β) (Figure 1E). The expression of CD80 and CD86 indicates the activation of pro-inflammatory macrophages and promotes their antigen presentation function [26–29]. We compared the expression of the costimulatory molecules CD80 and CD86 in LPS-induced and Mn^2+^-treated macrophages and observed that Mn^2+^ induced more robust transcriptional activity than LPS treatment (Figure 1F). To verify whether Mn^2+^ can regulate the phagocytosis of macrophages, we co-cultured BMDM with GFP tag with pHrodo-labeled tumor cells (ID8) for 24 h, and further observed the effect of macrophages on tumor cells by flow cytometry. The results showed that BMDM had almost no phagocytosis of tumor cells under normal conditions, while BMDM enhanced the phagocytosis of tumor cells after Mn^2+^ treatment (Figure 1G, 1H).

### Combined use of Mn^2+^ and IL-12 promotes T cell adaptive immune responses by macrophages *in vitro*

**Figure 2:**
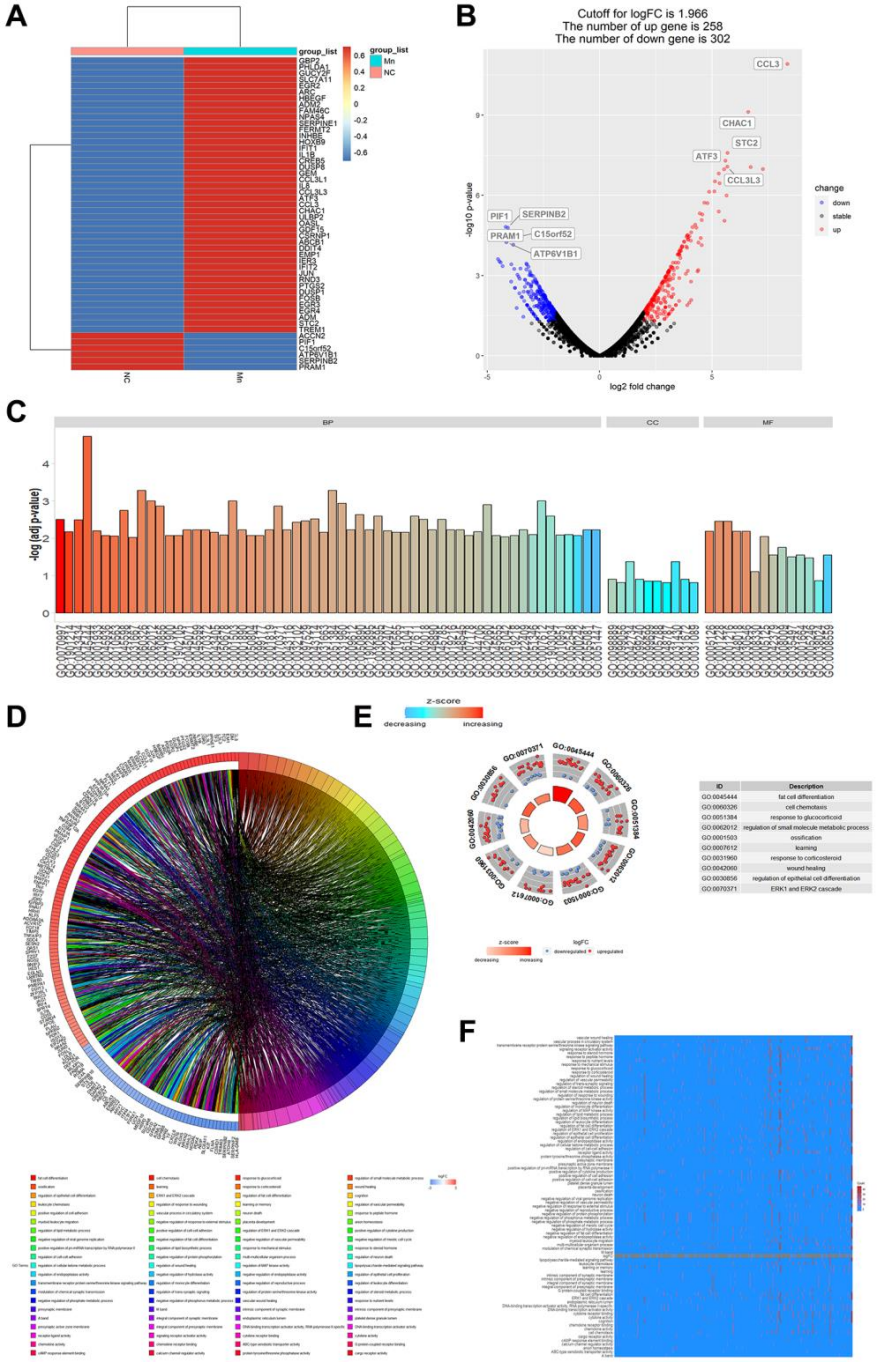
**Analysis of gene regulation by Mn^2+^ in macrophages.** (**A**) Heat map of cellular gene regulation after Mn^2+^ treatment. (**B**) Volcano plot of differentially expressed genes with a log-fold change (logarithmic value of differential gene expression) cut-off value of 1.966. (**C**) Histogram of GO enrichment terms. (**D**) Chord plot of enriched genes and their related pathways. (**E**) GO terms of upregulated and downregulated genes. (**F**) Heatmap of enrichment results.

RNA-seq data (GSE77216) of Mn^2+^-treated myeloid cells were used to analyze the regulatory effect of Mn^2+^ on gene transcription in macrophages. The analysis revealed that 258 genes were upregulated and 302 genes were downregulated by Mn^2+^ treatment compared with those in the control group, and Mn^2+^ treatment resulted in the increased expression of chemokines, inflammatory factors, and type I IFN-related genes (such as CCL3, IL1B, and IFIT1/2) (Figure 2A, 2B). GO enrichment analysis revealed that the differentially expressed genes were mainly enriched in GO terms such as cell chemotaxis (GO:0060326), regulation of small molecular, metabolic process (GO:0062012), presynaptic membrane (GO:0042734), epithelial membrane (GO:0005788), DNA binding transcription activator activity (GO:0001216), and cytokine activity (GO:0005125), which helped predict that Mn^2+^ could stimulate the activity of macrophages by altering cell adhesion, transcriptional regulation, and inflammatory factor release (Figure 2C–2F).

**Figure 3:**
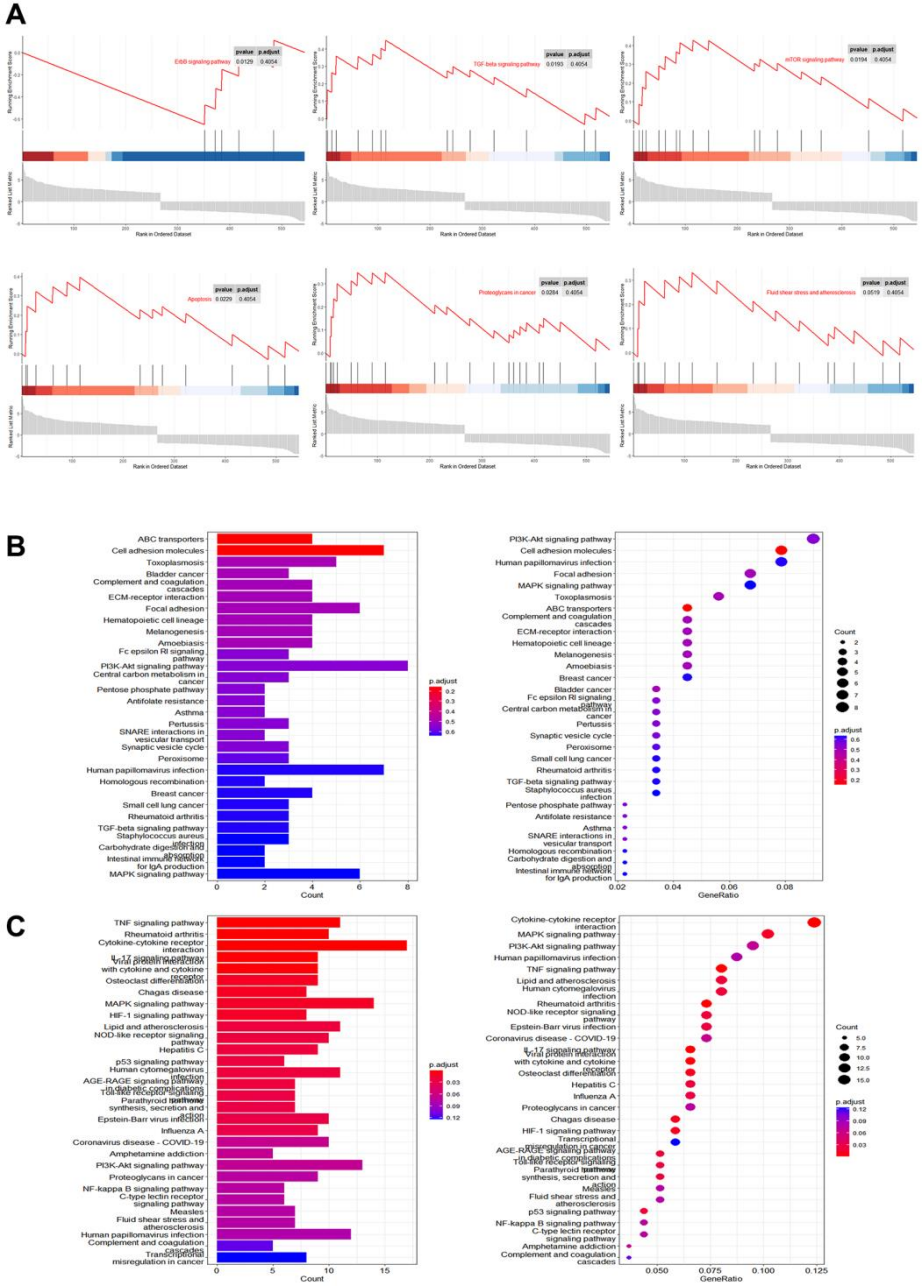
**Pathway analysis of gene regulation by Mn^2+^ in macrophages.** (**A**) GSEA of differentially expressed genes in Mn^2+^-treated group. (**B**) KEGG enrichment results of downregulated genes. (**C**) KEGG enrichment results of upregulated genes.

We evaluated the regulation of cellular pathways by Mn^2+^ by gene set enrichment analysis (GSEA). We observed that the differentially expressed genes were mainly enriched in the ErbB signaling pathway, TGF-β signaling pathway, mTOR signaling pathway, apoptosis, proteoglycans in cancer, fluid shear stress, and atherosclerosis (Figure 3A). KEGG enrichment analysis of downregulated (Figure 3B) and upregulated genes (Figure 3C) indicated that the upregulated genes were enriched in TNF-related pathways. These results suggest that Mn^2+^ may promote the activity of the proinflammatory M1 macrophage through the activation of innate immune pathways.

Antigen-presenting cells play a crucial role in linking innate and adaptive immunity by processing and presenting antigens to T cells. The enhanced phagocytic ability of tumor cells enables better initiation of antigen presentation. Mn^2+^ is a strong type I IFN stimulator that activates STING and its downstream pathways in the absence of infection. IL-12 is released by innate immune cells, such as macrophages, and responds to the release of IFN-γ with positive feedback. We hypothesized that the combined action of Mn^2+^ and IL-12 would promote adaptive immune responses.

**Figure 4:**
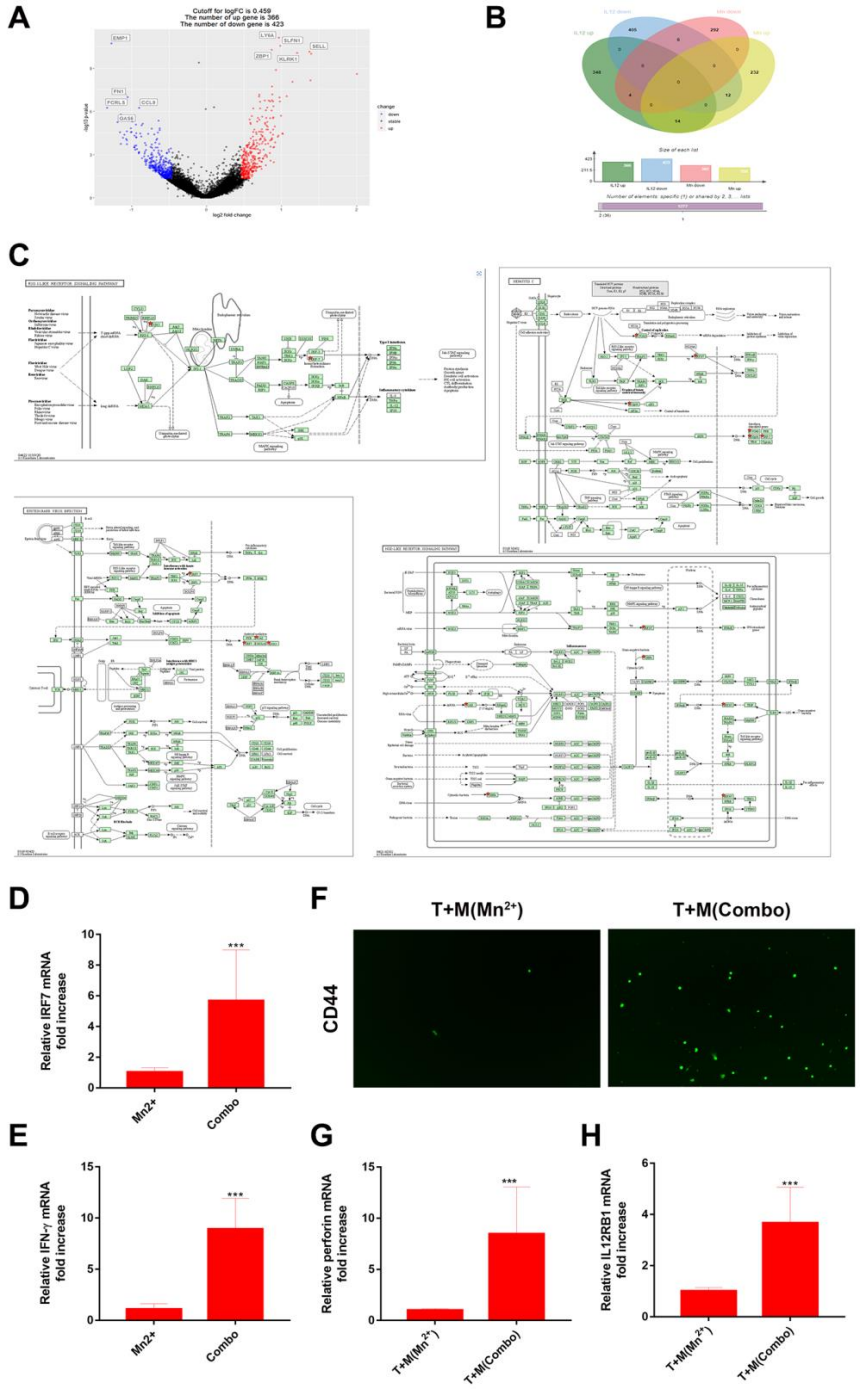
**Combined use of Mn^2+^ and IL-12 promoted adaptive immune responses of T cells by macrophages *in vitro*.** (**A**) Volcano plot of differential gene regulation by IL-12 in macrophages. (**B**) Venn diagram comparing regulated genes in Mn^2+^- and IL-12-treated macrophages. (**C**) Pathway diagram of KEGG enrichment results (red stars represent enriched genes). (**D**, **E**) qRT-PCR analysis of *IRF7* and IFN-γ gene expression in macrophages with different treatment conditions. (**F**) Immunofluorescence of CD44^+^ T cells after co-culture with macrophages treated under different conditions. (**G**, **H**) qRT-PCR analysis of perforin and IL-12Rβ1 genes. One representative experiment is displayed from at least three independent experiments, each performed in triplicate. Error bars represent SEM. Data were analyzed using unpaired *t*-test. Abbreviation: NS: not significant. ^*^P < 0.05, ^**^P < 0.01, ^***^P < 0.001.

We analyzed the RNA-seq data for IL-12 in tumor-associated macrophages (TAMs) (GSE136368). We compared the resulting differentially expressed genes (Figure 4A) with those of the Mn^2+^-treated cells and observed that the same genes, with 14 upregulated and six downregulated genes, were regulated for Mn^2+^ and IL-12 (Figure 4B). KEGG enrichment analysis revealed that the 14 upregulated genes were enriched in Hepatitis C, NOD-like receptor signaling, Epstein-Barr virus infection, and RIG-I-like receptor signaling pathways. Notably, *IRF7* was also enriched (Figure 4C). Cellular experiments were performed to verify this synergy in gene expression. The combination of Mn^2+^ and IL-12 increased the transcriptional activity of *IRF7* and IFN-γ production by macrophages (Figure 4D, 4E). The upregulation of IL-12Rβ1 is a conserved mechanism used by T cells to enhance sensitivity to IL-12 signaling [30], and T-cell receptor (TCR) stimulation enhances the expression of IL-12Rβ1 in CD8^+^ T cells. By coculturing T cells with Mn^2+^- and IL-12-treated macrophages engulfed with tumor cell debris under different conditions, we confirmed that the expression of the effector T marker CD44 was increased compared with that in the control group (Figure 4F), along with the transcriptional activity of the T-cell cytotoxicity-related perforin gene (Figure 4G) and expression of T cell IL-12Rβ1 (Figure 4H).

### Potent antitumor effect of Mn^2+^ Plus IL-12

**Figure 5:**
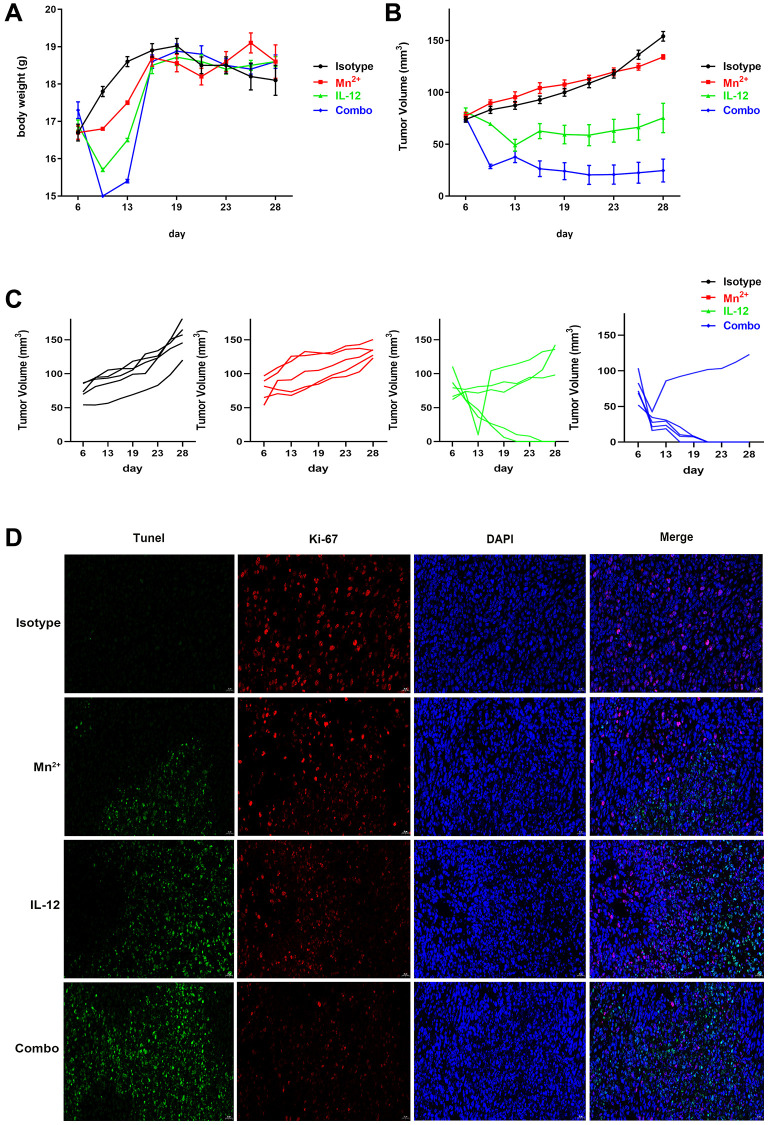
**Potent antitumor effect of Mn^2+^ and IL-12 combination treatment.** (**A**–**C**) Mice were subcutaneously inoculated with ID8 cells on day 0. Tumor-bearing mice were randomly divided into four groups: isotype control, Mn^2+^, IL-12, and a combination of Mn^2+^ and IL-12 (*n* = 5 mice per group). Treatment started on day 6. Body weight changes and tumor growth curves for each mouse are displayed. (**D**) Tumor tissues were fluorescently stained green, red, and blue to determine cell viability with TUNEL, with Ki-67, and to identify the nuclei, respectively (scale bar, 50 μm).

We evaluated the antitumor effect of Mn^2+^, IL-12, or a combination of Mn^2+^ and IL-12 (combination group) in a murine model of ovarian cancer. We observed no differences in mouse body weights between the treatment groups (Figure 5A). Furthermore, we demonstrated that the combination of Mn^2+^ and IL-12 had the most potent antitumor activity among the treatment strategies, based on the significant inhibition of tumor growth and burden (Figure 5B). Our results revealed that treatment with Mn^2+^, IL-12, or a combination of Mn^2+^ and IL-12 all suppressed tumor growth, with complete tumor regression observed in some mice treated with IL-12 alone or the combination treatment (Figure 5C), whereas the combination group achieved complete tumor regression in 4 of the five mice. Compared with the other treatment groups, the number of TUNEL-positive cells in the combination group was significantly increased. The expression of Ki-67, a cellular proliferation marker, was significantly decreased in tumors treated with a combination of Mn^2+^ and IL-12 (Figure 5D).

### Mn^2+^ and IL-12 combination treatment establishes an immune-supportive microenvironment

**Figure 6:**
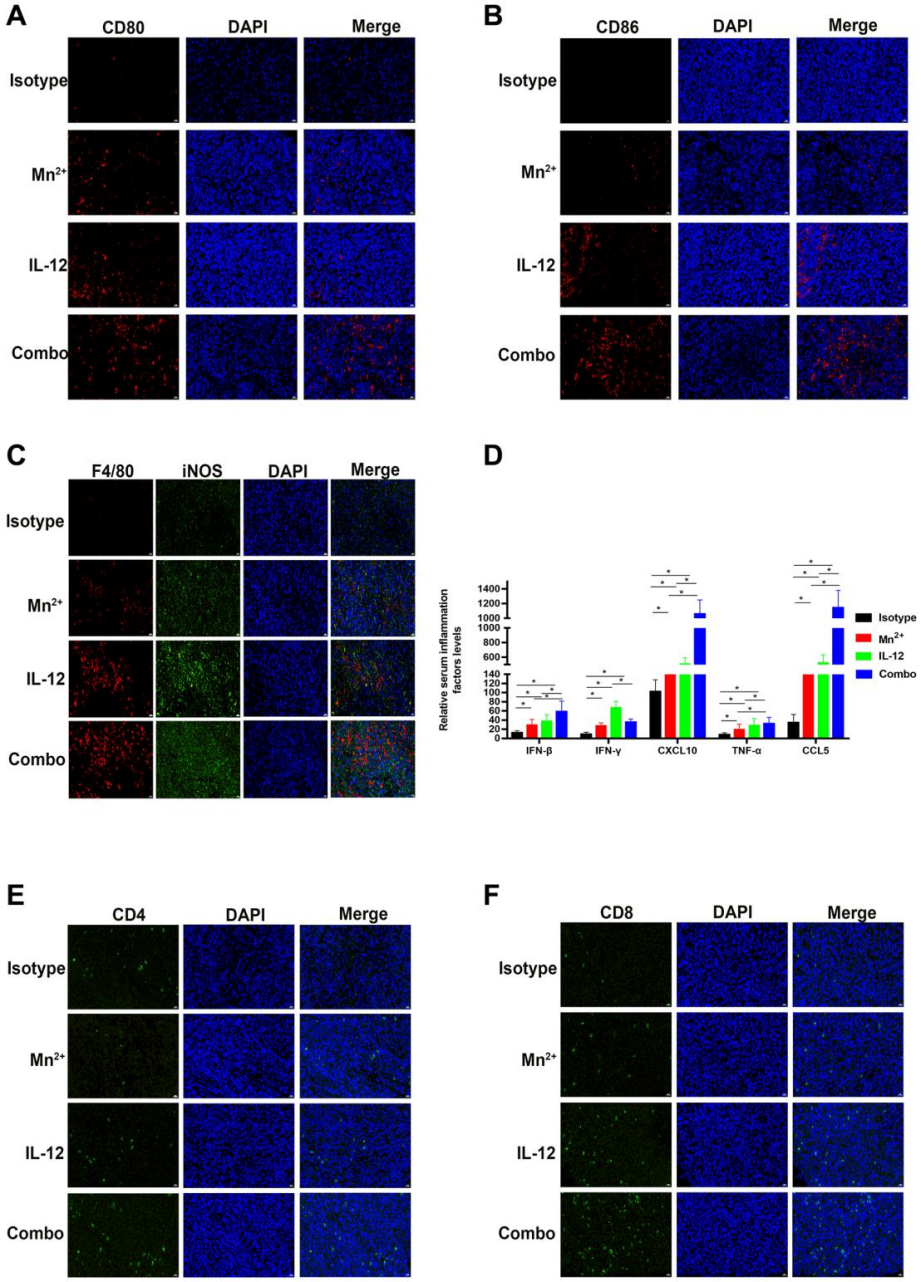
**Mn^2+^ and IL-12 combination treatment promoted the conversion of immune-exclusion tumors into immune-inflamed tumors and enhanced immune cell infiltration *in vivo*.** (**A**, **B**) Representative images of costimulatory CD80 and CD86 staining in the tumor center. (**C**) Representative images of M1-type macrophages co-localized by F4/80 (red) and iNOS (green) in the tumor center. (**D**) Cytokine levels in the circulation of tumor-bearing mice. (**E**, **F**) Representative images of CD4 and CD8 staining in the tumor center (scale bar, 50 μm). Abbreviation: NS: not significant. ^*^P < 0.05, ^**^P < 0.01, ^***^P < 0.001.

In the mouse tumor model, the levels of the macrophage co-stimulators CD80 and CD86 were higher in tumors of the combination group than in the Mn^2+^ and IL-12 alone groups (Figure 6A, 6B). The levels of M1-type macrophages were significantly increased in the combination group compared with that in the other individual therapy groups (Figure 6C). Compared with the isotype control group, increased levels of inflammatory factors and chemokines were observed in the circulation of mice in all three treatment groups to various degrees (Figure 6D). The expression of IFN-γ was significantly lower in the combination group compared with that in the IL-12 alone group. The number of infiltrating T cells in the center of the tumor also increased in the combination group (Figure 6E, 6F). The enhanced infiltration of CD4^+^/CD8^+^ T cells may have contributed to the improved cancer regression in the combination therapy group.

### Mn^2+^ and IL-12 combination treatment enhances persistent tumor resistance in mice

**Figure 7:**
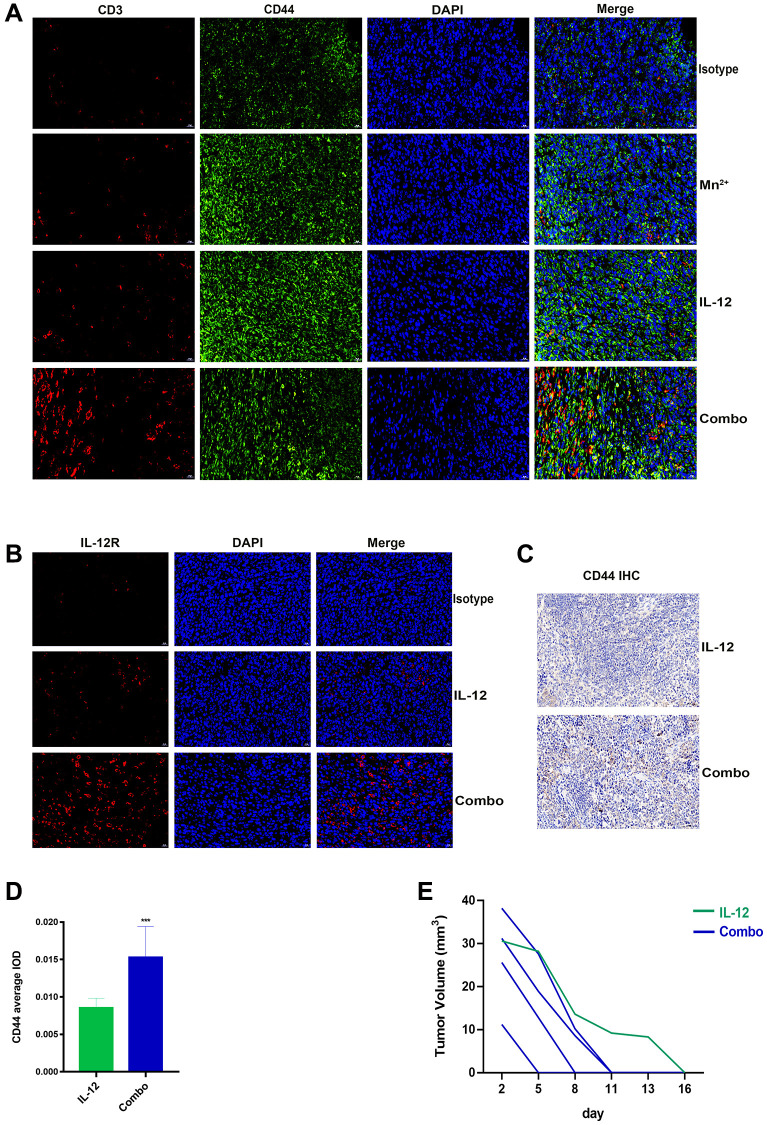
**Mn^2+^ and IL-12 combination treatment promoted sustained tumor resistance *in vivo*.** (**A**) Representative images of memory T cells co-localized by CD3 (red) and CD44 (green) in the tumor center. (**B**) Representative images of IL-12R expression in the tumor center. (**C**) Representative images of CD44 immunohistochemical staining of mouse spleens (scale bar, 50 μm). (**D**) Immunohistochemical quantification. (**E**) Tumor growth curves in tumor-bearing mice are displayed. Abbreviation: NS: not significant. ^*^P < 0.05, ^**^P < 0.01, ^***^P < 0.001.

Significantly more memory T cells were observed in the tumors of the combination group than in the Mn^2+^ and IL-12 alone groups (Figure 7A). IL-12R expression was significantly higher in the combination group compared with that in the IL-12 alone group (Figure 7B). Only mice in the IL-12 and combination groups exhibited complete tumor regression, possibly attributed to memory T cell homing to the spleen.

We performed histochemical staining of the spleens of tumor-ablated mice and observed that the combination treatment resulted in increased CD44 expression in mouse spleens (Figure 7C, 7D). To compare the antitumor capacity between the two treatment groups, we performed secondary tumor transplantation in mice with complete tumor regression (one died owing to human error). We observed that mice undergoing combination therapy exhibited faster tumor regression (5–11 d) (Figure 7E).

## DISCUSSION

In the TME, the STING pathway plays a crucial role in cross-presenting antigens and immune initiation. The development of STING agonists has been a hot topic in cancer treatment. However, traditional STING agonists, such as cGAMP, have shortcomings, such as low stability and poor transmembrane ability. Compared with newly developed STING agonists, Mn^2+^ has the advantages of low manufacturing costs and well-studied toxicity. As a natural and readily available STING agonist, Mn^2+^ may be a promising adjuvant for immunotherapy [17].

Manganese is a trace element involved in various physiological activities, including reproduction, development, energy metabolism, and antioxidant defense [16]. Mn also regulates innate immunity by enhancing the sensitivity of the STING pathway [15]. Notably, 2 μM Mn^2+^ conferred the antiviral ability to the human monocyte THP1 cell line *in vitro* [15]. Furthermore, Mn^2+^ treatment has high biological safety, with no toxicity related to excessive use [19]. In this study, we demonstrated that Mn^2+^ treatment promoted the proinflammatory phenotype and anti-tumor ability of macrophages. Mn^2+^ can independently stimulate STING activity to promote the type I IFN response in a manner independent of free DNA (Figure 1). Mn^2+^ can promote the phagocytosis of macrophages by increasing integrin expression and eliminating the immunosuppressive effect of CD47-overexpressing cancer cells [20]. Our experiments indicated that Mn^2+^ promoted the phagocytosis of tumor cells by macrophages. IL-12 signaling in T and NK cells induces the expression of IFN-γ, which could promote immunity. Through bioinformatic analysis, we observed that Mn^2+^ and IL-12 share a common regulatory mechanism. We also confirmed that Mn^2+^ combined with IL-12 could better promote the activation of effector T cells *in vitro*, which may be related to Mn^2+^ initiating macrophage antigen presentation to promote TCR and IL-12Rβ1 expression in T cells.

This novel cocktail therapy of Mn^2+^ and IL-12 exhibited more effective antitumor activity than monotherapies. Specifically, the combination therapy significantly improved the efficacy of IL-12, especially with respect to changes in the tumor immune environment. Combined therapy promoted M1 macrophage polarization, significantly increasing the density of infiltrating CD8^+^/CD4^+^ T cells in the tumor center. Furthermore, the combination therapy also promoted the sustained antitumor ability of mice, intratumoral invasion of CD44^+^ memory T cells, and spleen homing of T cells.

Systemic administration of IL-12 leads to toxicity associated with increased production of the inflammatory factor IFN-γ. The combination therapy had a lower IFN-γ cycle level than that of IL-12 alone (Figure 6D). This may be related to Mn^2+^ promoting macrophage phagocytosis of tumors and stimulating the TCR pathway of T cells through antigen presentation. IL-12 is expressed on immune cells through IL-12Rβ1 and IL-12Rβ2 receptor complexes emit signals. Upregulation of IL-12Rβ1 is a conserved mechanism for T cells to enhance sensitivity to IL-12 signaling [30]. TCR stimulation enhanced IL-12Rβ1 expression in CD8^+^ T cells above that of non-activated T and NK cells. Therefore, Mn^2+^ stimulates TCR expression in T cells by further increasing antigen presentation by promoting tumor phagocytosis by macrophages. TCR specific response of T cells to increase IL-12Rβ1 expression and increased sensitivity of T cells to IL-12 *in vivo*. Combination therapy can competitively bind IL-12 by promoting T cell TCR activation, thereby reducing the release of IFN-γ in the circulation. The ultimate goal is to reduce the adverse effects of IL-12.

**Figure 8:**
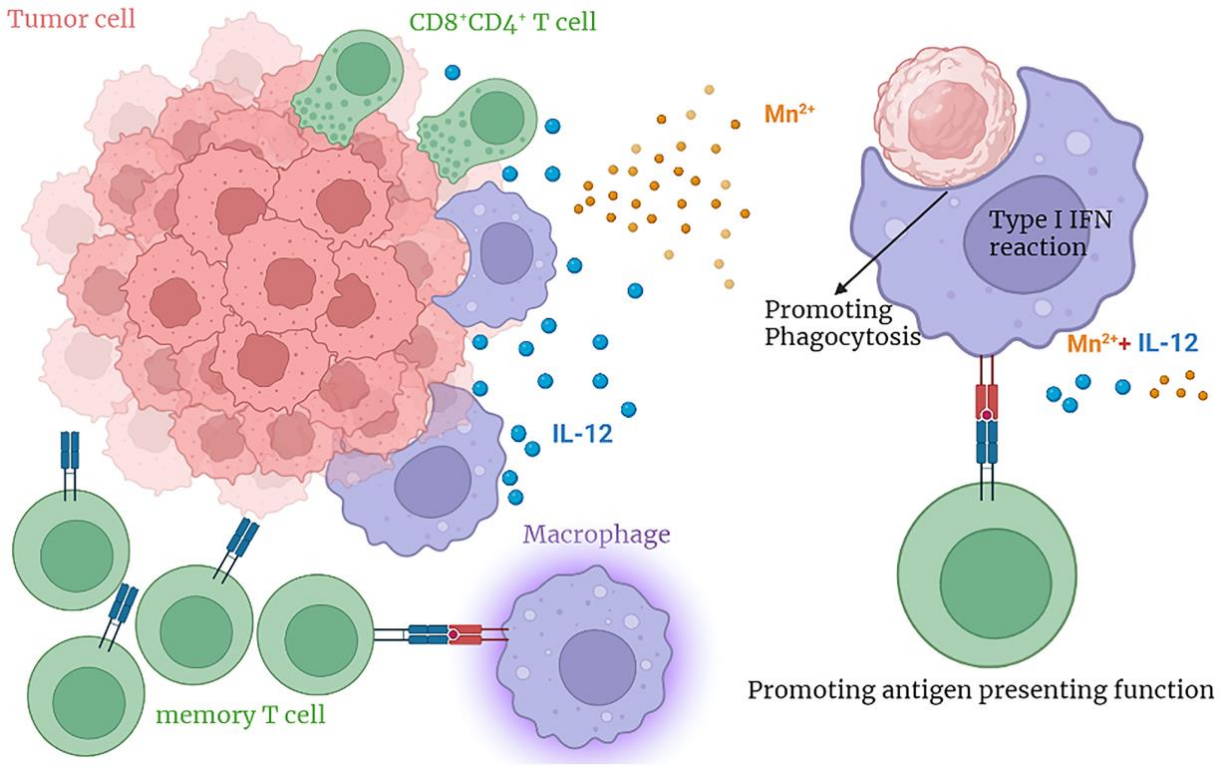
The graphical abstract.

In future studies, we will elucidate the mechanism by which the combined therapy exerts its antitumor effects and determine the optimal drug dose through *in vitro* and *in vivo* experiments to provide an efficient and safe approach that could be rapidly translated to clinical cancer treatment trials (Figure 8).
